# Human-Origin iPSC-Based Recellularization of Decellularized Whole Rat Livers

**DOI:** 10.3390/bioengineering9050219

**Published:** 2022-05-19

**Authors:** Aylin Acun, Ruben Oganesyan, Maria Jaramillo, Martin L. Yarmush, Basak E. Uygun

**Affiliations:** 1 Center for Engineering in Medicine and Surgery, Massachusetts General Hospital, Harvard Medical School, Shriners Hospitals for Children, Boston, MA 02114, USA; aacun@widener.edu (A.A.); oganesyan.rv@gmail.com (R.O.); maja983@gmail.com (M.J.); ireis@sbi.org (M.L.Y.); 2 Department of Surgery, Massachusetts General Hospital, Boston, MA 02114, USA; 3 Department of Biomedical Engineering, Widener University, Chester, PA 19013, USA; 4 Department of Biomedical Engineering, Rutgers University, Piscataway, NJ 08854, USA

**Keywords:** decellularization, liver bioengineering, iPSCs, recellularization

## Abstract

End-stage liver diseases lead to mortality of millions of patients, as the only treatment available is liver transplantation and donor scarcity means that patients have to wait long periods before receiving a new liver. In order to minimize donor organ scarcity, a promising bioengineering approach is to decellularize livers that do not qualify for transplantation. Through decellularization, these organs can be used as scaffolds for developing new functional organs. In this process, the original cells of the organ are removed and ideally should be replaced by patient-specific cells to eliminate the risk of immune rejection. Induced pluripotent stem cells (iPSCs) are ideal candidates for developing patient-specific organs, yet the maturity and functionality of iPSC-derived cells do not match those of primary cells. In this study, we introduced iPSCs into decellularized rat liver scaffolds prior to the start of differentiation into hepatic lineages to maximize the exposure of iPSCs to native liver matrices. Through exposure to the unique composition and native 3D organization of the liver microenvironment, as well as the more efficient perfusion culture throughout the differentiation process, iPSC differentiation into hepatocyte-like cells was enhanced. The resulting cells showed significantly higher expression of mature hepatocyte markers, including important CYP450 enzymes, along with lower expression of fetal markers, such as AFP. Importantly, the gene expression profile throughout the different stages of differentiation was more similar to native development. Our study shows that the native 3D liver microenvironment has a pivotal role to play in the development of human-origin hepatocyte-like cells with more mature characteristics.

## 1. Introduction

Late-stage liver diseases, such as cirrhosis, acute hepatitis, and liver cancer, were reported to lead to approximately 4% of all deaths globally in 2010 [1]. In such severe cases, the current gold-standard treatment is orthotopic liver transplantation. Although liver transplantation has high success rates and has been immensely improved since its first application in 1963 [2], many patients suffer from long waiting times as there is a large gap between the number of organs needed and the number of those that are available for transplantation. It was reported that in 2019 only 8896 patients received a new liver out of a total of 17,000 in need of a liver transplant, showing that 48% of the patients in the waiting list could not receive a new liver [3]. Alternative clinical approaches are being explored to reduce donor scarcity, such as split liver transplantation and living donor transplantation; however, these approaches alone are not enough to minimize the gap.

In order to increase the supply of livers available for transplantation, an approach that yields a high number of functional liver substitutes is needed. In the past decade, organ engineering through decellularization has emerged as a promising approach. This approach makes use of the extracellular matrix (ECM) scaffolds of native organs by removing all cellular materials from the organs [4,5]. The resulting scaffolds maintain the original overall shape and ultrastructure of the native organ. In addition, the makeup of the scaffold is not heavily altered, leading to the preservation of organ-specific ECM–cell signaling. An important consideration in translating this approach to clinical settings is to successfully recellularize these scaffolds to reobtain the native functions. Primary hepatocytes have been strongly preferred in such attempts; however, lack of patient-specificity, limited sources, and proliferation potential challenge their large-scale clinical translatability. As an alternative, induced pluripotent stem cells (iPSCs) provide a patient-specific, easily accessible, and expendable cell source [6]. The combination of decellularized natural scaffolds and patient-specific iPSCs would enable the development of readily available, tailor-made liver substitutes for patients.

The differentiation of iPSCs into hepatocyte-like cells has been demonstrated by several groups [7,8,9,10,11,12,13]. An important challenge in the clinical translation of iPSC-derived cells, however, is their immature phenotype, as indicated by significantly lower expression of mature hepatic markers, such as P450 enzymes, as well as higher expression of fetal markers, such as alpha fetoprotein (AFP), compared to primary hepatocytes. In attempts to induce maturation of these cells, our group [14] and others [15,16,17] have used decellularized liver matrices as substrates and showed an improvement in mature hepatic functions in response to interactions with the liver matrix. Importantly, our group has shown that if iPSCs interact with a liver matrix in a 2D culture setting, starting from the early stages of differentiation, the resulting hepatocyte-like cells have a more mature phenotype [14]. Recellularization of decellularized whole rat livers with iPSC-derived hepatocytes was shown in a study by Park et al., where fully differentiated cells were seeded using perfusion [17]. However, the effects of performing a full differentiation within a whole decellularized liver have not been shown.

In this study, we recellularized decellularized rat livers with undifferentiated iPSCs and performed the differentiation of hepatocytes using a perfusion bioreactor system (Supplementary Figure S1). We show that the 3D native liver matrix and perfusion culture conditions improved the differentiation efficiency, as evidenced by the drastic increase in the expression of markers specific to each stage of differentiation. We also showed that differentiation within the liver matrix leads to a more developmentally similar expression pattern throughout the differentiation compared to the gold-standard geltrex substrates and static culture conditions. In addition, we added a 7-day long maturation step to the differentiation process which yielded higher albumin and urea secretion, along with a significant decrease in AFP expression, showing the improved maturity of iPSC-origin livers.

## 2. Materials and Methods

### 2.1. Rat Liver Procurement and Decellularization

Livers of 3-month-old female F. Lewis rats (*N* = 18) were procured with cannulas attached to the portal vein in accordance with the Institutional Animal Care and Use Committee (IACUC) at Massachusetts General Hospital. Rat livers were decellularized using an adaptation of a previously reported protocol [18]. Briefly, the rat livers were attached via the portal vein cannula to a single-pass perfusion system composed of a peristaltic pump and a bubble trap and perfused with deionized (DI) water for 16 h. Following DI water perfusion, the livers were perfused with 0.1% sodium dodecyl sulfate (SDS) (Sigma-Aldrich, Burlington, MA, USA) for 24 h and with 0.2% and 0.5% SDS for 1 h each. Finally, the livers were washed with DI water and Triton X-100 (Sigma-Aldrich) for 1.5 h each and with PBS for 3 h. Throughout the decellularization process a constant flow rate of 1.6 mL/min was used. The decellularized livers were maintained in PBS at 4 °C until use.

The day before recellularization, the decellularized rat livers were sterilized using PBS supplemented with 0.1% (*v*/*v*) peracetic acid (Pfaltz and Bauer, Waterbury, CT, USA) and 4% (*v*/*v*) ethanol. Sterilization was performed by perfusing the liver with 50 mL of peracetic acid solution and incubation in the same solution for 3 h. Following this treatment, the livers were washed by perfusion with 50 mL of PBS followed by perfusion with 50 mL of PBS supplemented with 2% penicillin/streptomycin (Invitrogen, Waltham, MA, USA) and 2.5 µg/mL amphotericin B (Sigma-Aldrich), then incubated in this solution overnight until cell seeding.

### 2.2. Cell Culture

Human skin fibroblast-derived iPSC line hIPS-K3 cells were kindly provided by Dr. Stephen Duncan (Medical College of Wisconsin, Milwaukee, WI, USA). The cells were maintained in Geltrex™ (LDEV-Free Reduced Growth Factor Basement Membrane Matrix, Gibco, Waltham, MA, USA) coated culture flasks in mTeSR plus culture medium (Stemcell Technologies, Vancouver, BC, Canada) with daily media changes. Once the cells reached 80% confluency, they were collected using ReLeSR (Stemcell Technologies). Throughout the culture the pluripotency of the cells was examined by daily observation of the colony phenotype.

### 2.3. Recellularization of Decellularized Rat Livers

After sterilization, the decellularized rat livers were connected to a bioreactor system (Harvard Apparatus) (Supplementary Figure S2) through the portal vein cannula and perfused with mTeSR plus media supplemented with 10 µM ROCK inhibitor (Stemcell Technologies) for 30 min. Then, 15 × 10^6^ iPSCs in 4 mL mTeSR plus media were injected directly into the bubble trap and allowed to reach the liver through perfusion at 8 mL/min. At this point the flow was stopped and the liver was incubated statically for 15 min at RT. Then, the bioreactor system was maintained in an incubator (5% CO_2_, 37 °C) with perfusion at 0.1 mL/min flow rate for 1.5 h. The cell seeding steps were repeated 3 more times, reaching a total cell number of 60 × 10^6^ per liver. Once the last 1.5 h perfusion at 0.1 mL/min flow rate was completed, the flow rate was increased to 2 mL/min. The next day, the culture media was replaced with fresh mTeSR plus without ROCK inhibitor. The livers were maintained with mTeSR plus culture medium for a total of 4 days to induce proliferation of iPSCs prior to the initiation of differentiation, with media changes every other day.

### 2.4. Perfusion Differentiation of iPSCs in Decellularized Rat Livers

**Figure 1:**
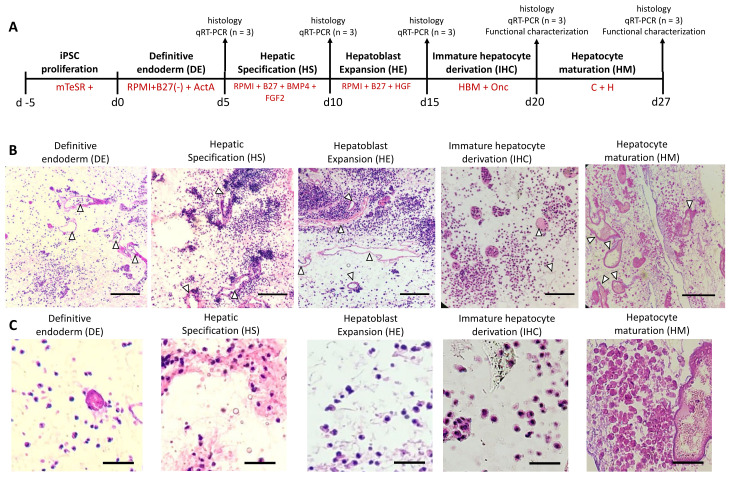
The perfusion differentiation of iPSCs to hepatocyte-like cells in rat livers. (**A**) The differentiation protocol and timeline showing the different analyses performed at each stage of differentiation. (**B**) Histological analysis of decellularized rat livers recellularized with iPSCs throughout the differentiation through H&E staining. White triangles show that vessels in the rat liver remained open following perfusion seeding and differentiation. (Scale bars = 200 µm.) (**C**) High magnification histological images showing cell morphology at different stages of differentiation. (Scale bars = 50 µm).

The differentiation of iPSCs to a hepatic lineage was achieved by adapting a previously established protocol [7] (Figure 1A). Briefly, differentiation was initiated by introducing RPMI (1640, Invitrogen) media supplemented with 50 ng/mL Activin A (ActA) (Peprotech, Cranbury, NJ, USA) and B27 without insulin (B27(-)) (Invitrogen) for 5 days. During the first 2 days, the media was also supplemented with 10 ng/mL bone morphogenic protein 4 (BMP4) (Peprotech) and 20 ng/mL fibroblast growth factor 2 (FGF-2) (Peprotech). The first 5 days of differentiation aimed to drive definitive endoderm (DE). Next, the media in the bioreactor was replaced with RPMI containing B27 with insulin supplemented with 20 ng/mL BMP4 (Peprotech) and 10 ng/mL FGF-2 (Peprotech) for 5 days for hepatic specification (HS). Over the following 5 days, the media was replaced with RPMI containing B27 with insulin supplemented with 20 ng/mL hepatocyte growth factor (HGF) (Peprotech) in RPMI/B27 to achieve hepatoblast expansion (HE). Finally, for 5 days the liver was perfused with Hepatocyte Basal Media (HBM) (Lonza, Rockville, MD, USA) supplemented with SingleQuots (without EGF) supplemented with 20 ng/mL Oncostatin-M (Onc) (R&D Systems, Minneapolis, MN) to achieve immature hepatocyte derivation (IHC). In order to induce a more mature phenotype of iPSC-derived cells, we added another step, namely, hepatocyte maturation (HM), by which the livers were perfused with hepatocyte growth media (C + H) (DMEM supplemented with 10% fetal bovine serum, 0.5 U/mL insulin, 7 ng/mL glucagon, 20 ng/mL epidermal growth factor, 7.5 μg/mL hydrocortisone, 200 U/mL penicillin/streptomycin, and 50 μg/mL gentamycin) for 7 days. Throughout the differentiation the bioreactor was oxygenated and maintained at 37 °C supplemented with 5% CO_2_ with daily media changes.

### 2.5. Quantitative Real Time PCR (qRT-PCR)

In order to determine the mRNA expression levels of specific markers at each stage of differentiation, livers were sacrificed at the end of each stage and approximately 2/3 of the livers were used for RNA extraction (*n* = 3 per stage). The liver tissues were flash frozen in liquid nitrogen and ground using a mortar and pestle. The resulting tissue was then used for RNA isolation using a PureLink RNA isolation kit (Thermo Fisher Scientific, Waltham, MA, USA), following the manufacturer’s instructions. The resulting RNA was used for cDNA synthesis using an iScript cDNA synthesis kit (Bio-Rad, Hercules, CA, USA), following the manufacturer’s instructions. The cDNA was then used in qRT-PCR analysis using a ViiA7 Real time PCR system (Thermo Fisher Scientific) and a power SYBR Green PCR master mix kit (Thermo Fisher Scientific), according to the manufacturer’s instructions. The list of primers used is provided in Supplementary Table S1. All expression levels were normalized to GAPDH expression. Results for pluripotency and endoderm marker expression were represented relative to undifferentiated iPSCs cultured on geltrex-coated well plates. Results for early and mature hepatic marker expression were represented relative to cells differentiated on geltrex-coated well plates unless stated otherwise.

### 2.6. Albumin and Urea Quantification

For albumin and urea quantification, media were collected at the end of the IHC stage and daily during the HM stage. For all experiments, the same volume of media was collected (10 mL). The level of albumin secreted in the livers was determined using a Human Albumin ELISA kit (Abcam, Cambridge, UK), following the manufacturer’s instructions. The urea nitrogen direct kit (Stanbio, Boerne, TX, USA) was used, following the manufacturer’s instructions, to determine the amount of urea secreted. Both albumin and urea contents were represented as micrograms secreted per liver.

### 2.7. Histological Analysis and Immunohistochemistry

For histological analysis, the recellularized rat liver tissues were collected at the end of each stage of differentiation and fixed with 10% formalin for 24 to 48 h at room temperature (RT) and then maintained in 70% ethanol at 4 °C. The tissues were then dehydrated and embedded in paraffin. The tissues were microsectioned to 5 µm thick slices and stained with hematoxylin (Leica, Wetzlar, Germany) and eosin (Leica) (H&E) to visualize the ECM and cell nuclei. The stained sections were imaged using a Nikon Eclipse E800 (Tokyo, Japan).

Immunohistochemistry analysis was performed at the Histopathology Research Core at Massachusetts General Hospital. Briefly, the recellularized rat liver tissues were embedded in Tissue-Tek O.C.T. compound (Sakura Finetek, Torrance, CA, USA) and frozen at −80 °C. The tissues were cryo-sectioned and labeled with antibodies against HNF-4a (HNF4A Monoclonal Antibody (F.674.9), Thermo Fisher Scientific), AFP (AFP monoclonal antibody (35436), Thermo Fisher Scientific), and Ki67 (Ki-67 Monoclonal Antibody (SolA15), eBioscience). The sections were then labeled with species-appropriate secondary antibodies as well as DAPI and imaged using an Olympus Nanozoomer slide scanner at 488 nm.

The quantitative analysis of HNF-4a, AFP, and Ki67 expression was performed by measuring the fluorescence intensity of the respective immunohistochemistry images. For the purpose of the analysis, the backgrounds of the extracellular matrices in the images were removed, using the same noise settings for each target, by means of ImageJ software (Image J 1.51). The results are represented as fluorescence intensity, arbitrary units (A.U.).

### 2.8. Statistical Analysis

For all statistical analyses Microsoft Excel Office 365 (Version 16.39, Redmond, WA, USA) and GraphPad Prism (Version 8.3.1, San Diego, CA, USA) were used. The Student’s *t*-test with Welch’s correction and one-way-ANOVA analysis were used, and statistical difference was defined as *p* < 0.05. All results are represented as averages ± standard deviation of 3 different liver recellularization experiments using the same cell line but different cultures within passages 21–27.

## 3. Results

### 3.1. iPSCs Attach to and Differentiate within Decellularized Rat Livers

In order to develop a functional and patient-specific liver, the use of human-origin iPSC-derived cells is a promising approach. We used a closed bioreactor system to recellularize the decellularized rat livers (Supplementary Figure S2A,B). The iPSCs were introduced into the parenchymas of the livers in four steps through the portal veins. The cells were maintained for five days prior to differentiation to allow for cell proliferation. At the end of the first stage of differentiation, we observed that the cells penetrated the parenchymas of the livers, with minimal numbers of cells remaining in the vasculature. With the start of differentiation, we sacrificed the livers at the end of each stage of differentiation and assessed cell localization and changes in cell number qualitatively (Figure 1B). We observed that until the HE stage there was an increase in cell number, as shown by histological analysis, which stabilized after this stage. We also observed macroscopically that the livers appeared opaquer at the end of the differentiation period compared to immediately after decellularization, showing that the cells populated the parenchymas uniformly throughout the livers (Supplementary Figure S3A,B).

### 3.2. iPSC Differentiation Efficiency Is Induced by the Native Liver Microenvironment

**Figure 2:**
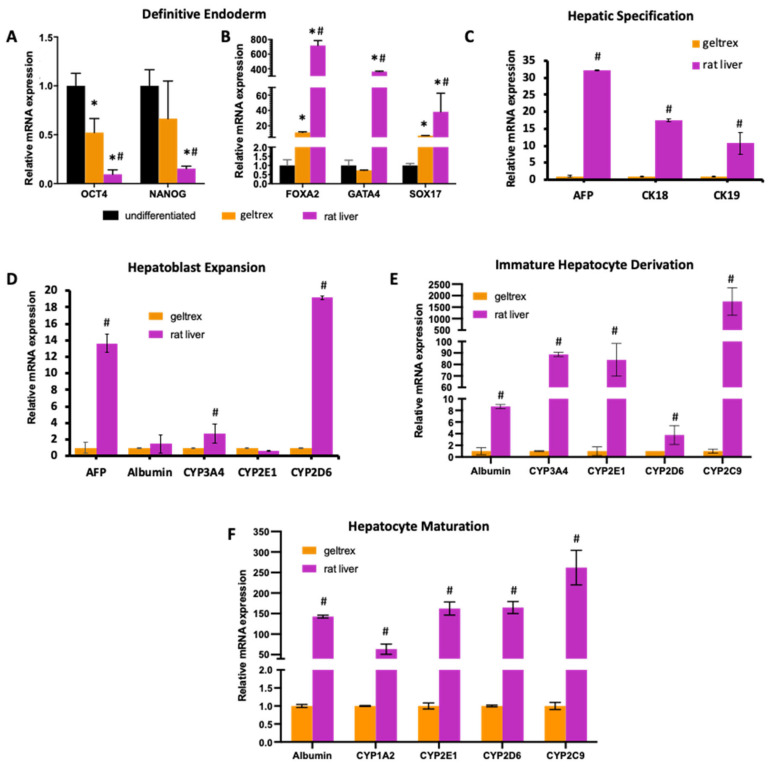
mRNA expression levels of key markers specific to each differentiation stage. qRT-PCR analysis showing mRNA expression of (**A**) pluripotency and (**B**) endoderm markers at the DE stage. (**C**) mRNA expression of early hepatic markers at the HS stage. mRNA expression of mature hepatic markers at (**D**) the HE stage, (**E**) the IHC stage, and (**F**) the HM stage, relative to undifferentiated iPSCs. (* indicates statistically significant differences compared to undifferentiated iPSCs (*p* < 0.05); ^#^ indicates statistically significant differences in the decellularized rat liver group compared to the geltrex group (*p* < 0.05).) For rat liver data, samples from three scaffolds at DE, HS, HE, IHC, and IHC+ stages were analyzed. For geltrex data, at least three different differentiations at each stage were used in the analysis.

Following the attachment and proliferation of iPSCs within decellularized rat livers, we started the differentiation process, following a previously established protocol [7]. We determined the expression of pluripotency, endoderm, developmental hepatoblast, and mature hepatocyte markers at the corresponding stages throughout the differentiation. We compared the results to cells differentiated on conventional geltrex-coated culture well plates and normalized all results to undifferentiated iPSCs (Figure 2A,B; Supplementary Figure S4A–D) or to cells differentiated on geltrex (Figure 2C–F). We observed that with the induction of the endoderm lineage, the expression of pluripotency marker OCT-4 significantly decreased in both geltrex and decellularized rat liver groups (Figure 2A). The decrease in expression of NANOG was significant in decellularized rat livers compared to undifferentiated iPSCs; however, the decrease in the geltrex group was not significant (*p* = 0.24). In addition, the expression of the endoderm-specific markers SOX17 and FOXA2 significantly increased in both groups. Importantly, using decellularized rat livers as substrates significantly improved the endoderm induction efficiency compared to differentiation on geltrex, as shown by significantly higher expressions of SOX17 (*p* = 0.015), GATA4 (*p* < 0.0001), and FOXA2 (*p* < 0.001) (Figure 2B), revealing the effects of native liver matrices at early stages of differentiation.

At the hepatic specification stage, we observed that the markers expressed during early development of the liver, namely, AFP, CK18, and CK19, were induced in both the geltrex and decellularized rat liver groups. Expression of these markers was significantly higher in the decellularized rat liver group compared to the geltrex group (AFP: 32.2 ± 0.03 fold, CK18: 17.4 ± 0.3 fold, and CK19: 10.7 ± 3.2 fold increase) (Figure 2C). Undifferentiated iPSCs showed no expression of these hepatic markers (Supplementary Figure S4A).

At the end of the hepatoblast expansion stage of differentiation, we determined the expression of both developmental and mature hepatocyte markers. The expression of AFP was 13.6 ± 1.1-fold higher in the decellularized rat livers compared to the geltrex group (*p* < 0.001) (Figure 2D). In addition, expression levels of CYP3A4 and CYP2D6 were significantly higher in decellularized rat livers compared to the geltrex group (*p* = 0.026 for CYP3A4, and *p* < 0.0001 for CYP2D6), whereas the expressions of albumin and CYP2E1 were similar.

We compared the expression of only mature hepatocyte markers at the last two stages of differentiation (Figure 2E,F). The most significant difference in expression between the decellularized rat livers and the geltrex group was observed in these stages. For each marker, the expression levels were significantly higher in the decellularized rat livers. At the IHC stage, the expression of CYP2C9 was over 1745 ± 603-fold higher in decellularized rat livers compared to the geltrex group (Figure 2E). In addition, the expression levels of CYP3A4 and CYP2E1 showed an over 80-fold difference between geltrex and decellularized rat livers (CYP3A4:88.7 ± 1.8 and CYP2E1: 84 ± 14.2). With the completion of the hepatocyte maturation step, the drastic difference in the expression of mature hepatocyte markers was maintained (Figure 2F). The expression levels of albumin, CYP1A2, CYP2E1, CYP2D6, and CYP2C9 in decellularized rat livers were, respectively, 142.6 ± 3.3-, 63.3 ± 12.4-, 162.3 ± 15.9-, 164.7 ± 14.6-, and 262.1 ± 42.1-fold higher, showing the importance of the contribution of the native liver environment to the late stages of differentiation. We did not observe any significant expression of the mature hepatic markers in the undifferentiated cells, as expected (Supplementary Figure S4B–D).

### 3.3. Differentiation within Decellularized Rat Livers Better Mimics Native Development

**Figure 3:**
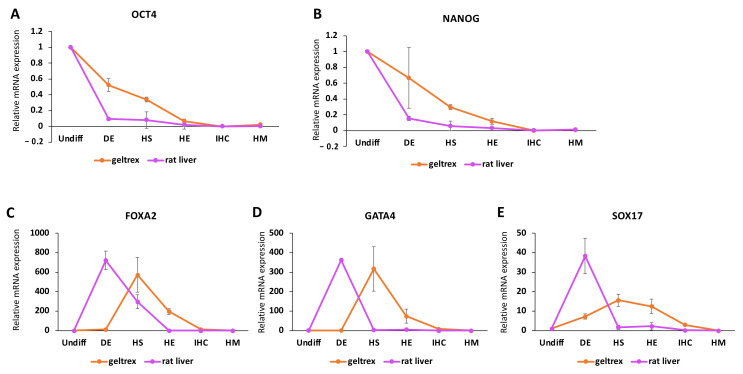
Changes in the expression patterns of pluripotency and endoderm markers in iPSCs throughout differentiation in native liver ECM. qRT−PCR analysis results showing the mRNA expression levels of (**A**) OCT4, (**B**) NANOG, (**C**) FOXA2, (**D**) GATA4, and (**E**) SOX17 relative to the respective expression levels in undifferentiated iPSCs throughout the differentiation in the geltrex and decellularized rat liver (rat liver) groups. For rat liver data, samples from three scaffolds at the DE, HS, HE, IHC, and IHC+ stages were analyzed. For geltrex data, at least three different differentiations at each stage were used in the analysis.

In order to assess the role of native liver micro- and macroenvironments on iPSC differentiation, we determined the changes in expression trends of some key markers throughout the differentiation process. We observed that the expression of the pluripotency markers OCT4 and NANOG decreased significantly at the start of differentiation within rat livers; however, there was a more gradual decrease when iPSCs were differentiated on geltrex (Figure 3A,B). Within decellularized rat livers, the lowest levels of OCT4 and NANOG expression were reached at the DE stage and maintained at similar levels throughout. In the geltrex group, the lowest expression for the markers was achieved at the HE stage, with OCT4 expression gradually decreasing at each stage and NANOG expression remaining constant during DE and showing a significant drop in the HS stage, followed by a milder decrease in the HE stage. When we traced the expression of the endoderm markers SOX17, GATA4, and FOXA2 in iPSCs differentiated in decellularized rat livers, we observed that the highest expression level for each marker was reached in the DE stage, as expected (Figure 3C–E). In the geltrex samples, however, the peak for each marker was observed in the HS stage. The peak values for GATA4 and FOXA2 in decellularized rat liver and geltrex samples did not show any significant differences (Figure 3D,E). The peak expression levels for SOX17, however, were significantly higher (*p* = 0.015) in decellularized rat livers (38.3 ± 9.1) compared to geltrex samples (15.5 ± 3.1) (Figure 3C).

**Figure 4:**
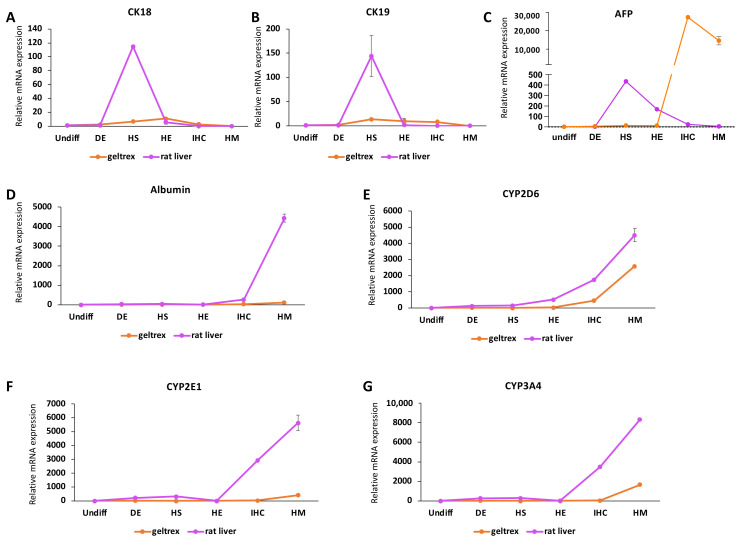
The changes in expression patterns of early and mature hepatic markers in iPSCs throughout differentiation in native liver ECM. qRT−PCR analysis results showing the mRNA expression levels of (**A**) CK18, (**B**) CK19, (**C**) AFP, (**D**) albumin, (**E**) CYP2D6, (**F**) CYP2E1, and (**G**) CYP3A4 relative to the respective expression levels in undifferentiated iPSCs throughout the differentiation in the geltrex and decellularized rat liver (rat liver) groups. For rat liver data, samples from three scaffolds at the DE, HS, HE, IHC, and IHC+ stages were analyzed. For geltrex data, at least three different differentiations at each stage were used in analysis.

We determined expression patterns during early hepatic specification through tracking the expression levels of CK18, CK19, and AFP. Both CK18 and CK19 reached their highest expression levels at the HS stage in decellularized rat livers (Figure 4A,B). In geltrex samples, the highest expressions of CK18 and CK19 were reached at the HE and HS stages, respectively. For both markers the peak expression was significantly higher (*p* < 0.001) in decellularized rat livers (CK18: 114.7 ± 2.1, CK19: 144.1 ± 42.4) compared to geltrex (CK18: 10.7 ± 3.9, CK19: 13.4 ± 0.7). The expression of AFP, however, showed a different trend, with the highest values being recorded in the IHC stage in the geltrex group and in the HS stage in decellularized rat livers (Figure 4C). In addition to the delayed increase in expression, the peak values reached in the geltrex group (27,394 ± 3254) were significantly higher (*p* < 0.001) compared to the decellularized rat livers (435.3 ± 0.4).

Finally, we determined the expression patterns of the mature hepatic markers albumin, CYP2D6, CYP2E1, and CYP3A4 (Figure 4D–G). All of these markers followed a similar trend, showing increased expression at the later stages of differentiation. For all markers, peak expression levels were recorded at the end of the HM stage regardless of the cell attachment substrate. In the decellularized rat livers, however, the highest expression levels reached were significantly higher compared to the highest levels reached in the geltrex samples (albumin: *p* < 0.001, CYP2D6: *p* = 0.004, CYP2E1: *p* < 0.001, CYP3A4: *p* < 0.001) Interestingly, the expression levels of CYP2D6, CYP2E1, and CYP3A4 started to increase in the HS stage in decellularized rat livers, while this response was delayed until the IHC stage in the geltrex group.

### 3.4. A Functional Human iPSC-Derived Hepatocyte-Based Liver Is Developed

**Figure 5:**
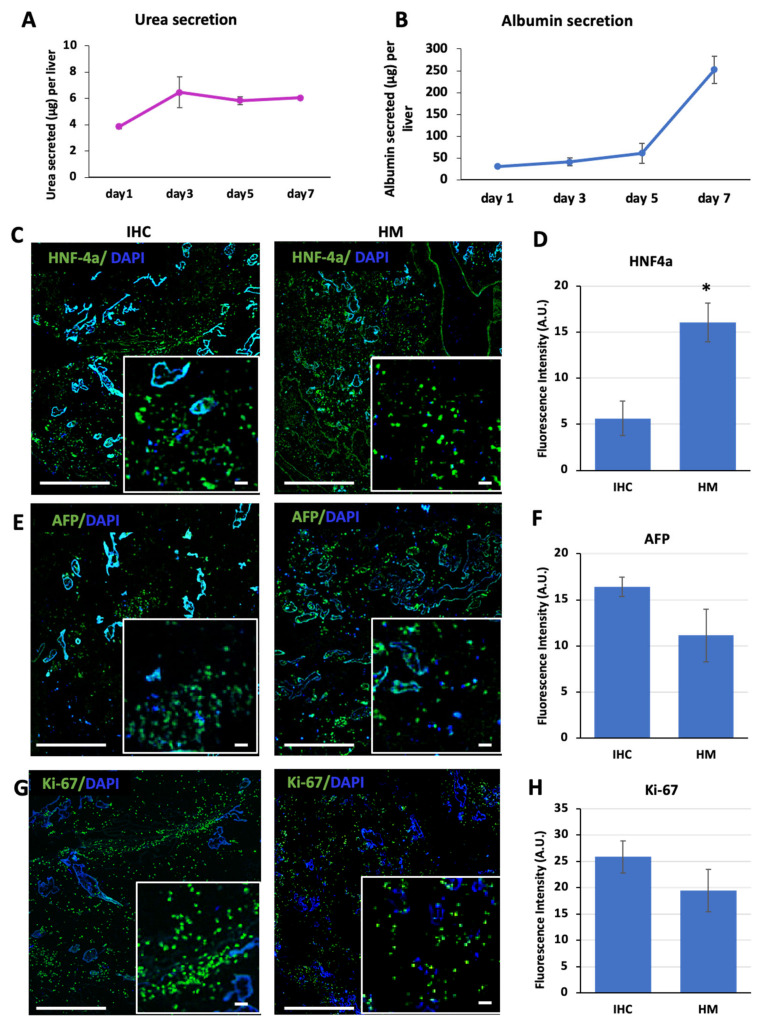
The functional characterization of recellularized rat livers. The amounts of (**A**) urea and (**B**) albumin secreted by the recellularized livers determined on days 1, 3, 5, and 7 were determined for the HM stage. Images of the immunohistochemistry analysis for (**C**) HNF4a, (**E**) AFP, and (**G**) Ki-67 in recellularized rat livers in the IHC and HM stages. Fluorescence intensity quantification of the immunohistochemistry images showing changes in the expression levels of (**D**) HNF4a, (**F**) AFP, and (**H**) Ki-67 in the IHC and HM stages. Three scaffolds at the IHC stage and three scaffolds in the IHC+ stages were analyzed for quantification. (* indicates statistically significant differences (*p* < 0.05); scale bars = 200 µm; scale bars for insets = 50 µm.)

We determined the functional maturation of the livers at the protein level by assessing the albumin and urea levels secreted by the iPSC-derived hepatocytes during the HM stage (Figure 5A,B). We collected media samples from the bioreactors at days 1, 3, 5, and 7 of culture during the HM stage. Our results showed a significant increase in both urea and albumin secretion over 7 days compared to day 1 (*p* = 0.002 for urea, *p* < 0.001 for albumin). The urea secretion was 3.8 ± 0.2 µg/mL on day 1 of the HM stage, whereas it reached 6.0 ± 0.1 µg/mL on day 7 (Figure 5A). The albumin secretion on day 1 of the HM stage was 31 ± 3 µg per liver and increased to 252 ± 31 µg per liver at the end of the HM stage. The increase in urea secretion followed a different trend compared to albumin secretion such that the increase in urea secretion peaked on day 3 of culture followed by a stabilization over the following days. The albumin secretion, however, showed a gradual increase in the early days of the HM stage, while increasing significantly from day 5 of culture (Figure 5B).

In addition, we determined the protein expression of HNF4a, AFP, and Ki-67 in the livers at the end of the IHC and HM stages through immunohistochemistry (Figure 5C–G). We observed that the iPSC-derived hepatocyte-like cells were positive for all markers at both the IHC and HM stages. The expression of HNF4a showed a significant increase (*p* = 0.034), indicating that the maturation stage induced more mature cells (Figure 5C,D). In addition, there was a decrease in AFP protein levels at the HM stage compared to the IHC stage in decellularized rat livers, although this decrease was not significant (*p* = 0.133) (Figure 5E,F). This decrease was in line with the decrease observed in AFP mRNA expression levels (Figure 4C). The mRNA expression of AFP was 24.2 ± 16.7 at the IHC stage and 5.1 ± 0.5 at the HM stage, showing a decrease with maturation, although the difference was not significant (*p* = 0.124). We also determined the expression of the proliferation marker Ki-67. In response to the maturation stage, we observed a slight decrease in Ki-67 levels; however, this decrease was not significant (*p* = 0.215) (Figure 5G,H).

## 4. Discussion

The use of decellularized scaffolds in clinic is a promising alternative to replacing damaged or diseased tissues and organs. The successful decellularization of rodent to human livers has been shown by our group and others [18,19,20,21]. The next challenge in carrying this approach to clinic is the recellularization of these native scaffolds with patient-specific cells to develop functional tissues/organs. iPSCs, since their discovery, have been the main focus for personalized treatments. Although promising, the current differentiation protocols yield hepatocyte-like cells with fetal-like phenotypes, leading to inferior functionality compared to primary cells [22]. To tackle this problem our group and others have used native liver scaffolds as differentiation substrates and demonstrated improved phenotypes. Wang and colleagues [16] cultured iPSC-hepatocytes on 500 µm thick decellularized rat liver ECM discs and used a poly-l-lactic acid (PLLA)–collagen mix as a control. They observed that the liver ECM induced higher mRNA expression of P450 enzymes and higher albumin secretion compared to the controls. After 14 days of culture on the liver matrix, expression levels of the fetal markers AFP and CYP3A7 were significantly lower compared to controls, showing improved maturity in response to ECM–cell interactions. In another study, Park et al. [17] investigated the effect of porcine liver matrices on porcine iPSC-derived hepatocyte-like cells. For this, they supplemented the differentiation medium with solubilized porcine liver matrix. The highest albumin expression was achieved when the supplementation was performed at the last stage of differentiation. In addition, they seeded the median lobes of decellularized rat livers with porcine iPSC-derived cells via perfusion. They showed that there was albumin and urea secretion by day 3 of culture, yet the apoptosis rate significantly increased by day 7 of culture in the recellularized scaffolds. In a previous study, our group differentiated human iPSCs on human decellularized liver matrix and determined the stage of differentiation at which plating the cells on human liver matrix would yield the best differentiation results [14]. The results showed that exposing the cells to liver matrix from the earliest stage of differentiation induced higher expression of the markers specific to each stage at the respective stages. Guided by these findings, we investigated how differentiation would be affected by the 3D native liver environment under perfusion culture, showin—for the first time, to our knowledge—full differentiation within the whole decellularized rat livers.

Rat livers were selected as our platform as their smaller size and wider availability rendered them suitable for our proof-of-concept study. It is important to minimize the presence of residual detergents in decellularized scaffolds due to their cytotoxicity. Different methods of residual SDS removal from decellularized scaffolds have been shown [23] and in this study we have performed an extensive wash to remove residual detergent presence. We have adapted a well-studied method for decellularizing rat livers in this study. Since the successful outcome of the adapted method has been shown numerous times in our previous studies [24,25,26], we do not provide here a detailed characterization of the decellularization process. The iPSCs reached the parenchymas of the livers and attached to the liver matrices in agreement with reports showing iPSC attachment to 2D gels made of liver matrix [14,17]. Histological analysis showed that the livers were more heavily populated at the HS and HE stages, suggesting growth in cell number at earlier stages of differentiation, as well as further confirming that there was no cytotoxicity due to residual detergents.

ECM–cell interactions have been shown to have an important role in cell differentiation and function in various tissues [27,28]. The specific effect of decellularized liver matrices on the change of expression patterns in iPSCs throughout their differentiation towards hepatic lineages, as well as their effect on differentiated cell functions [14,16,17], have been shown in 2D settings. However, the structural organization and other advantages of a 3D microenvironment are overlooked in such 2D settings and the direct effect of the 3D native liver environment has not been shown. In one study, when differentiated using a sandwich culture method with human decellularized matrix, iPSC expression patterns showed higher expression of markers specific to each stage compared to cells differentiated on Matrigel [14]. At the definitive endoderm stage, the endoderm markers GATA-4, FOXA2, and SOX17 were expressed at significantly higher levels compared to the Matrigel groups. However, in this culture setting the difference was 10-fold or less. In our study, the differentiation within whole decellularized rat livers yielded an over 65-fold increase in FOXA2 and an over 500-fold increase in GATA4 expression compared to the geltrex samples. These drastic differences were observed at the later stages of differentiation in the expression levels of mature hepatocyte markers. Overall, the induction of expression of markers at this level was greater than the increase reported for 2D liver matrix substrates, indicating the importance of the 3D microenvironment in addition to the components of the ECM. This drastic increase was likely induced by a combination of factors, including the increased cell–ECM interactions provided in the whole liver setting, the ECM remaining intact without going through a digestion step that was used to develop the 2D substrates, and the perfusion culture delivering fresh and oxygenated media continuously. Our results are consistent with those of Sassi et al. [29], who showed that the perfusion culture of primary human hepatocytes in a bioreactor system induced significantly higher cell viability and functioning compared to static cultures within the same scaffolds.

In addition to comparing the expression of markers at the respective differentiation stages at which they are expected to peak, we also observed the expression patterns throughout the differentiation so as to understand the overall expression changes in the 3D environment. Interestingly, we observed an immediate decrease in pluripotency markers in decellularized rat livers, while a more gradual decrease took place in the geltrex group. This could potentially point to a lower differentiation efficiency in the geltrex group compared to the decellularized rat livers. This is in line with the previous observations of Jaramillo et al., who reported that decreases in the expression of OCT4 and NANOG were more gradual when cells were differentiated on Matrigel surfaces compared to 2D human liver matrix gels [14]. We also observed that the endoderm markers SOX17, GATA4, and FOXA2 reached peak expression levels at the HS stage in the geltrex group as opposed to the DE stage in decellularized rat livers. A similar expression trend was noted in the work of Jaramillo et al., although it was reported for FOXA2 expression only. In the next stage of differentiation, we observed large differences in the expression levels of the developmental hepatic markers CK18 and CK19 between the decellularized rat liver and geltrex groups. The delayed increase in the expression of endoderm markers is in line with a lower efficiency in the induction of hepatic markers at the HS stage.

A commonly used hepatic differentiation protocol consists of four stages that constitute a 20 day-long protocol [7]. Just as is observed in hepatocyte differentiation, other cell lineages also face the problem of resulting iPSC-derived cells exhibiting fetal expression patterns and associated inferior functionality [30,31]. The 3D environment and the native liver microenvironment are two factors that are shown to be effective in improving the mature phenotypes of cells [16,32,33]. Another factor was shown to be an extended culture with appropriate culture media [34,35]. In this study we added a final stage that we named hepatocyte maturation, in which the cells were cultured for an extra 7 days with a medium used for primary hepatocytes. We hypothesized that this additional step, along with the 3D native microenvironment and physiologically relevant flow conditions, would contribute to further maturation of the iPSC-derived hepatocyte-like cells. Although we did not observe any changes in the organizational structure of hepatocytes within the parenchymas of the livers histologically, at mRNA or protein levels the cells showed higher expression of mature markers, including important CYP450 enzymes, albumin, and HNF4a [36], after the maturation step compared to the IHC stage. The albumin secretion achieved at the end of the HM stage was superior to other reports, such as Park et al.’s, in which it was reported that about 4 µg albumin per liver was secreted, while above 250 µg was secreted through our protocol. In addition, Sassi et al. reported approximately 40 µg albumin secretion in the lateral left lobe of rat livers populated with primary human hepatocytes after 11 days or longer of culture under perfusion [29]. It should be noted that in the study by Park et al. only the median lobes of livers were populated and that the cells interacted with decellularized liver ECMs only after full differentiation. Similarly, in the study by Sassi et al., only the left lateral lobes of rat livers were used and characterized for albumin secretion under perfusion. Even though the albumin secretion analysis in our study showed superior results, the urea secretion recorded by Sassi et al. in the left lateral lobes of primary hepatocyte-populated rat livers was higher. Overall, although inferior compared to those for native liver functions and primary hepatocyte urea secretion levels, our results still suggest an improvement compared to other reports of iPSC-hepatocyte-populated livers, with potential benefits owing to cell–ECM interactions provided throughout the differentiation process. Feldhoff et al. reported albumin secretion levels for a male Sprague Dawley rat of 540 µg per g of liver per hour [37], which is much higher than the levels reached in our setting. Through improving the number of cells seeded in decellularized livers, both albumin and urea synthesis rates can be improved.

An important observation we made was that expression of the fetal marker AFP was significantly lower following the maturation stage. This is in line with the natural development of the liver, as AFP expression has been reported to be higher in the fetal liver compared to the adult liver [36]. This decrease suggests a more mature-like expression profile. The observation of a similar trend in the geltrex group further suggests that the HM stage added here induced maturation of iPSC-hepatocytes and that it is important to include it in regular practices. Another indicator of maturity in hepatocytes is quiescence. In the healthy adult liver, the proliferation capacity of hepatocytes is extremely low as they are quiescent [38]. The proliferation of hepatocytes is only triggered by injury in the mature state [39]. Although not significant, we observed a slight decrease in Ki67-positive cells after the maturation stage. Taken together, the increase in the expression of mature hepatocyte markers, the increases in albumin and urea secretion, and the decreases in AFP and Ki67 expression suggest an improved maturity evoked by the 3D microenvironment and the maturation stage that we incorporated into our protocol.

## 5. Conclusions

Overall, we have reported, for the first time, the perfusion differentiation of iPSCs within whole decellularized rat livers. In this report, we have shown the importance of the native 3D microenvironment in developing human-origin hepatocyte-like cells with more mature characteristics and the possibility of further improving hepatic functioning through prolonged perfusion culture. We believe that although the native liver microenvironment used here is of rat origin, the results are clinically relevant, as we have shown in our previous studies that rat and human decellularized liver matrices have similar contents [21]. We emphasize that further optimization and improvements are required, as well as the addition of non-parenchymal liver cells, to produce tissues of clinically applicable sizes and functionalities and suggest that the native microenvironment and structural organization are crucial to achieve this.

## Data Availability

The data presented in this study are available on request from the corresponding author.

## Supplementary Materials

The following supporting information can be downloaded at: https://www.mdpi.com/article/10.3390/bioengineering9050219/s1, Figure S1: Schematic representation of the study; Figure S2: (A) The bioreactor system used for recellularization of decellularized rat livers. (B) The inside of the bioreactor chamber with a rat liver being perfused with culture media prior to cell seeding; Figure S3: (A) The rat liver at the end of decellularization. (B) The picture of a recellularized rat liver at the end of IHC stage; Figure S4: The mRNA expression levels of key markers specific to early and mature hepatic markers as normalized to expression in undifferentiated iPSCs; Table S1: Primers for qRT-PCR.
