# Supplementary material for: Human-Origin iPSC-Based Recellularization of Decellularized Whole Rat Livers
Source: Bioengineering (Basel). 2022 May 19;9(5):219. doi: 10.3390/bioengineering9050219 (PMC9137624; doi:10.3390/bioengineering9050219)
Supplement: Supplementary file 1 [file bioengineering-09-00219-s001.zip › bioengineering-1728321-supplementary.pdf]

Supplemental Information
for
Human-origin iPSC-based recellularization of decellularized whole rat livers

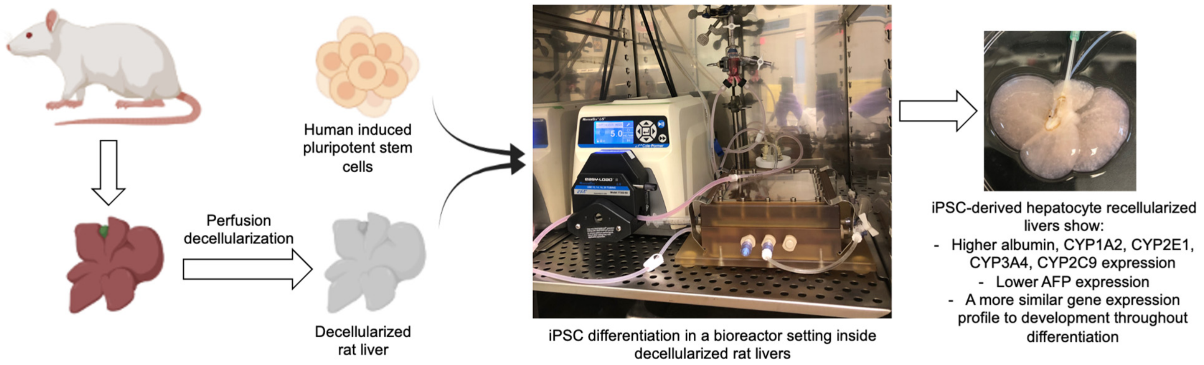

Supplementary Figure S1. Schematic representation of the study. Rat livers were decellularized and populated with undifferentiated human iPSCs in a bioreactor system. Within the bioreactor the iPSCs were differentiated to hepatocytes using a 20-day long protocol, followed by a 7-day maturation step, under prefusion culture. The recellularized livers were characterized at different stages of differentiation to show mRNA expression patterns throughout compared cells differentiated on geltrex. In addition, the functionality of the livers were determined at the end of maturation step.

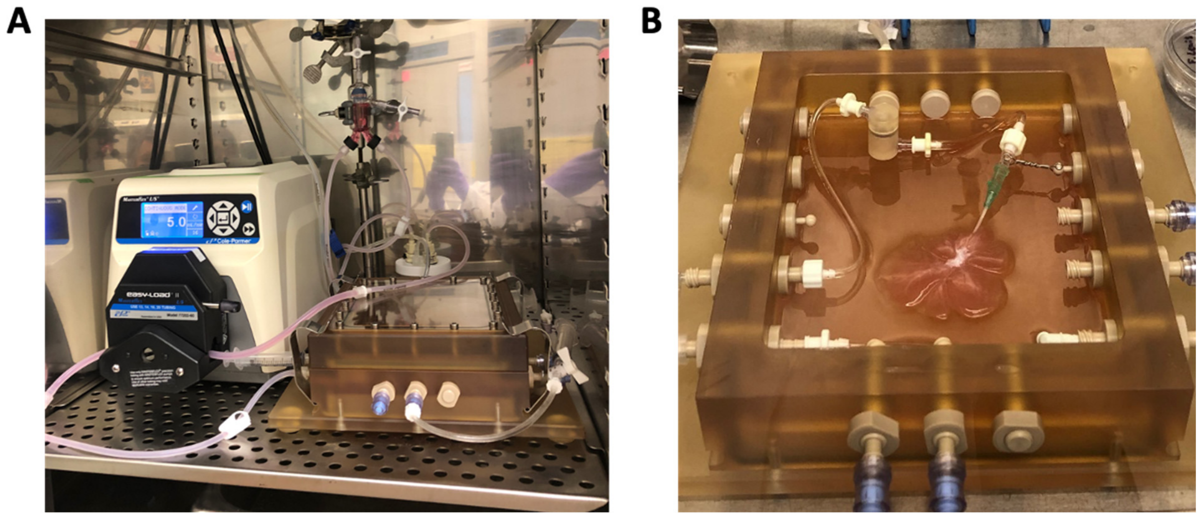

Supplementary Figure S2. (**A**) The bioreactor system used for recellularization of decellularized rat livers. (**B**) The inside of the bioreactor chamber with a rat liver being perfused with culture media prior to cell seeding.

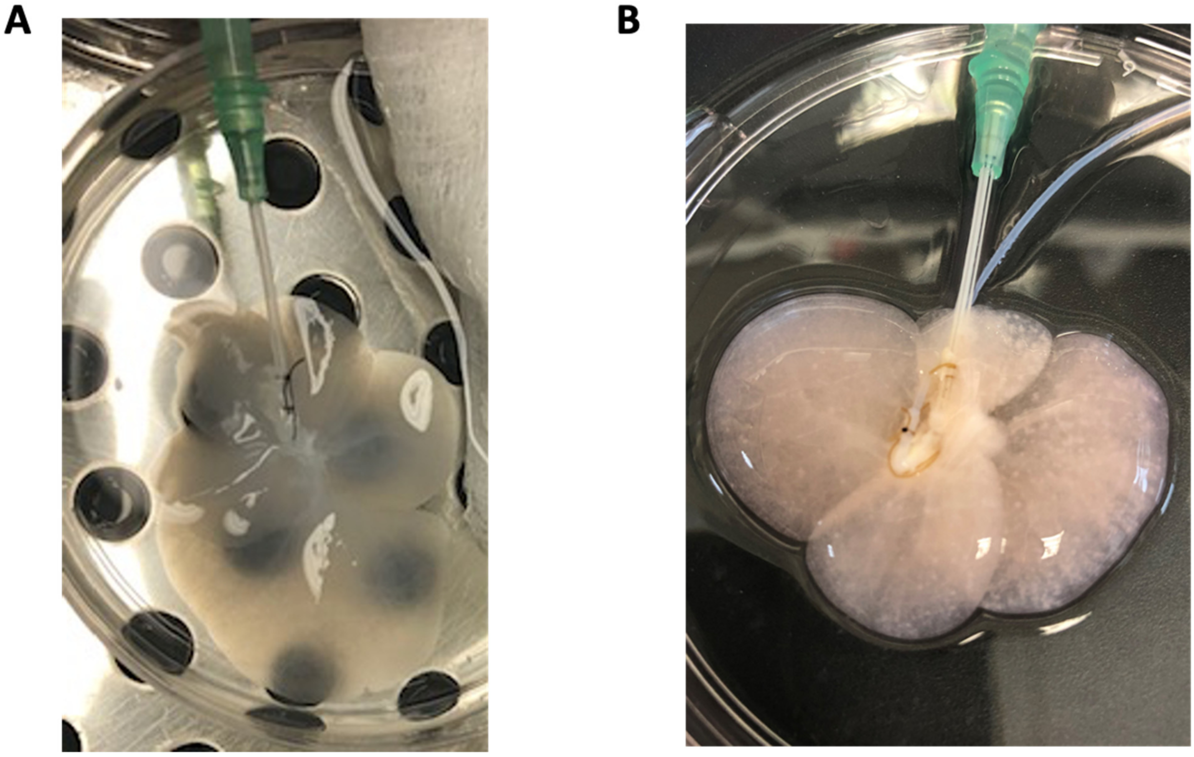

Supplementary Figure S3. (**A**) The rat liver at the end of decellularization. (**B**) The picture of a recellularized rat liver at the end of IHC stage.

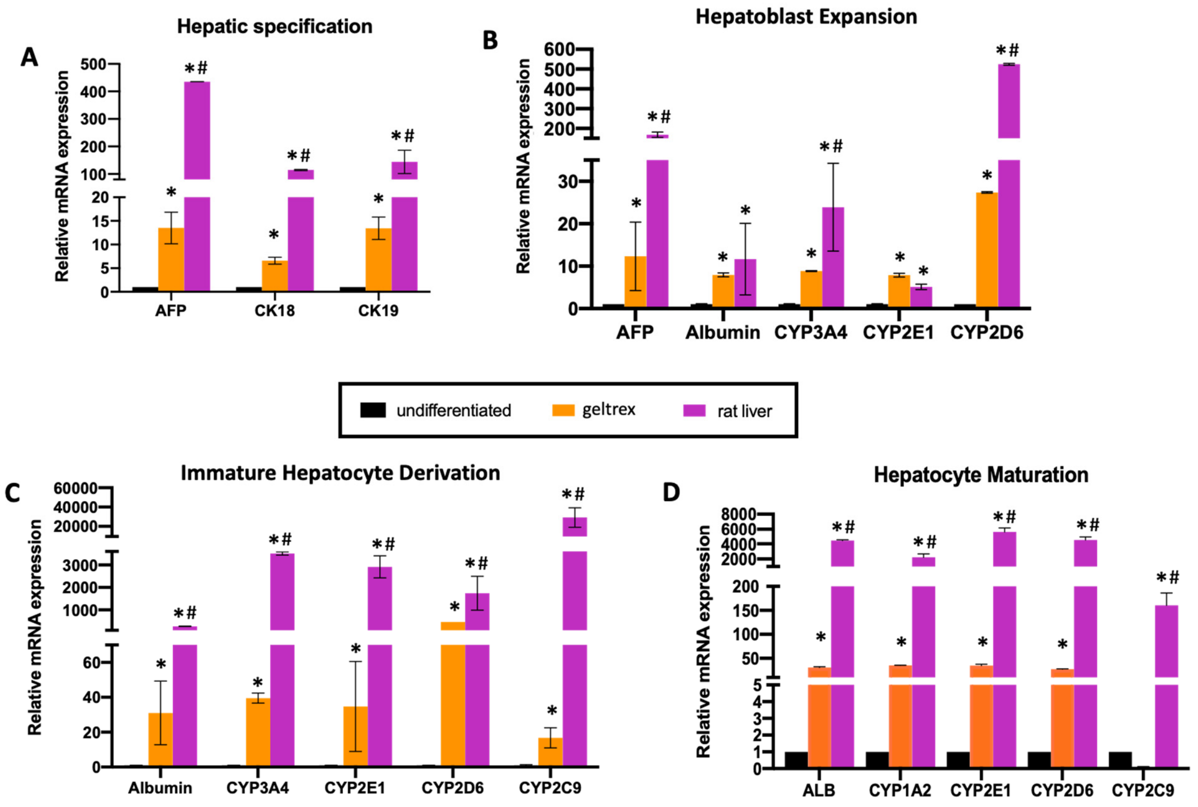

Supplementary Figure S4. The mRNA expression levels of key markers specific to early and mature hepatic markers as normalized to expression in undifferentiated iPSCs. The qRT-PCR analysis showing the mRNA expression of (**A**) early hepatic markers at HS stage, and the mRNA expression of mature hepatic markers at (**B**) HE stage, (**C**) IHC stage, and (**D**) HM stage, relative to undifferentiated iPSCs. (* indicates statistically significant difference compared to undifferentiated iPSCs ($p<0.05$) and $^{\#}$ represents statistically significant difference in decellularized rat liver group compared to geltrex group ($p<0.05$))

Supplementary Table S1. Primers for qRT-PCR

**ALBUMIN**	TCTTCTGTCAACCCCACACG	GCAACCTCACTCTTGTGTGC
**CYP1A2**	CTTCTTCCCCATCCTTCGCTA	AGGAACCTCTGGTTGAAGGC
**CYP2E1**	GGTGAGTGTTAGAGTTACTTGAGGA	TAGAGGTTAGAGCTGCCCCT
**CYP2D6**	AGCACTTCAGCTTCTCGGTG	GCACAAAGCTCATAGGGGGA
**CYP3A4**	CTTCTTCCCCATCCTTCGCTA	AGGAACCTCTGGTTGAAGGC
**CYP2C8**	ACTACCTCATCCCCAAGGGC	TTGGATTAGGAAATTCTTTGTCATCA
**GAPDH**	AATGGGCAGCCGTTAGGAAA	GCCCAATACGACCAAATCAGAG
**AFP**	CAGGGTGTTTAGAAAACCAGCTAC	TGCTGGAGTGGGCTTTTTGT
**CK18**	GAGGGCTCAGATCTTCGCAA	CCAGCTGCAGTCGTGTGATA
**CK19**	TGGAGATGCAGATCGAAGGC	CTCAGCGTACTGATTTCCTCCT
**OCT-4**	CGAGAAGGATGTGGTCCGAG	TGTGCATAGTCGCTGCTTGA
**NANOG**	CAATGGTGTGACGCAGGGAT	TGCACCAGGTCTGAGTGTTC
**FOXA2**	ATTTCTTCTCCCTTGCGTCT	CTCTTAAGAAGACGACGGCT
**GATA4**	GTAGTGAGATGACAGGCCAG	GGATCCAAACCAGAAAACGG
**SOX17**	CGCACGGAATTTGAACAGTA	GGATCAGGGACCTGTCACAC
